# Mechanical Properties and Crack Propagation Behavior of Defective Cement Mortar Reinforced with Hybrid Steel–Carbon Fibers

**DOI:** 10.3390/ma19143101

**Published:** 2026-07-19

**Authors:** Gaozhen Hu, Liang Li, Anhua Xu, Yuanji Li, Chenchen Zhang, Shiren La

**Affiliations:** 1 School of Civil and Transportation Engineering, Qinghai Minzu University, Xining 810007, China; hgz9801@163.com (G.H.); xahua@qhmu.edu.cn (A.X.); 2 Qinghai Provincial Key Laboratory of Tibet Plateau Highway Construction Maintenance Technology, Xining 810003, China; 18297138810@163.com (L.L.); qhjiaotongkeji@163.com (Y.L.); 13856797409@163.com (C.Z.); 3 Qinghai Lake Tourism Development Group Co, Ltd., Xining 810003, China; 4 School of Water Conservancy and Civil Engineering, Qinghai Vocational and Technical University, Xining 810016, China

**Keywords:** digital image correlation, steel–carbon fiber cement mortar, mechanical properties, tension-compression strength ratio, failure pattern, crack resistance performance

## Abstract

**Highlights:**

**What are the main findings?**

- The compressive strength of the double-hole specimens (6.70 MPa) in the reference group (NM) was 28.6% lower than that of the single-hole specimens (9.38 MPa), and the crack propagation time under compressive loading was shortened from 92 s to 53 s, and from 19 s to 12 s under tensile loading, indicating that multiple defects significantly weaken the mechanical properties and accelerate brittle failure.
- In the double-hole specimens of the reference group, a herringbone-shaped strain concentration band formed between the two holes and led to penetration failure; hybrid fiber incorporation (e.g., S2C02) markedly weakened the strain concentration band between the holes, expanded the extent of high-strain zones while their peak values decreased, and shifted the failure mode from concentrated penetration to distributed multi-crack development.

**What are the implications of the main findings?**

- From the perspectives of mesoscopic strain and macroscopic mechanics, the different reinforcement mechanisms of steel fibers and carbon fibers under defective conditions and their combined effect are revealed. This not only enriches the theory of fiber-reinforced cementitious materials but also provides new insights for the study of the mechanical behavior of defective cement mortar in engineering practice.
- It is clarified that fiber reinforcement has an optimal content, so as to avoid performance degradation and cost waste caused by blind addition, thereby providing data support for the performance optimization and engineering selection of fiber-reinforced cementitious materials.
- The incorporation of fibers weakened the strain concentration in the specimens, reduced the crack propagation velocity, and changed the failure mode of some specimens from brittle to quasi-ductile; this provides a certain reference for improving the crack resistance of cement-based materials, delaying crack penetration, and enhancing their service performance.

**Abstract:**

To enhance the crack resistance and toughness of cement mortar, steel fibers (SF), carbon fibers (CF), and their hybrid combinations were incorporated at various volume fractions. Cubic specimens with single or double prefabricated holes were tested using compressive and splitting tensile tests combined with digital image correlation technology (DIC). to evaluate mechanical properties, crack propagation, and failure patterns. Results indicate that steel fibers primarily suppressed macrocracks via bridging and improved strength, while carbon fibers inhibited microcrack initiation and promoted uniform strain distribution. Hybrid fibers achieved combined reinforcement, with the S2C02 mixture exhibiting the best overall performance: compressive strength increased by 39.7–55.1%, tensile strength by 70.45–75.9%, and the toughness and tensile/compressive ratio were enhanced. DIC analysis showed reduced strain concentration, more uniform strain fields, delayed crack propagation, and a transition from brittle to quasi-ductile failure. These findings demonstrate an optimal hybrid fiber dosage and reveal the combined mechanism of steel–carbon fibers in defect-containing cement mortar, providing guidance for material design and performance optimization.

## 1. Introduction

Cement mortar materials are widely used in various engineering fields due to their ease of construction, wide availability of raw materials, and low cost, making them one of the most consumed and extensively applied civil engineering materials at present [1,2,3]. However, the ordinary cement mortar has its own limitations, such as high porosity, low tensile strength, poor toughness, and susceptibility to cracking [4,5]. Under the long-term action of the external environment and loading, cement mortar is highly prone to cracking. Once cracks initiate and propagate, the cracks will further develop over time, compromising the structural stability and durability, and leading to a significant reduction in its service life [6,7].

To overcome the inherent deficiencies of cement mortar, numerous researchers have carried out extensive studies from various aspects [8,9,10,11]. Relevant studies have shown that fiber incorporation has been proven effective in improving the mechanical performance and crack resistance of cementitious materials, enhancing the mechanical properties of the material [12,13], and inhibiting crack propagation [14,15,16]. Huang et al. [17] incorporated glass fiber polymer into mortar. Through mechanical testing, computed tomography (CT), and scanning electron microscopy (SEM) analysis, the results showed that the fibers significantly improved the mechanical properties of the mortar and reduced shrinkage. Małek et al. [18] evaluated the effect of glass fiber dosage on mortar performance and found that glass fiber incorporation significantly improved its mechanical properties. At an optimal dosage of 1800 g/m^3^, the compressive, flexural, and splitting tensile strengths increased by 31.5%, 29.9%, and 97.6%, respectively, relative to the control specimen. Liu et al. [19] conducted a series of mechanical tests, including compression, tension, and four-point bending, to investigate the effects of steel fibers and polyvinyl alcohol (PVA) fibers on concrete performance. The results indicated that the two fiber types influenced the microstructure in different ways, leading to distinct macroscopic mechanical behaviors.

Numerous studies have demonstrated that fiber reinforcement is an effective approach for improving the mechanical properties and crack resistance of cementitious materials. However, excessive PVA fiber incorporation resulted in a reduction in strength-related properties. Ma et al. [20] incorporated basalt fibers and carbon fibers into cement-based materials and reported that the flexural strength increased with increasing fiber content. In addition, carbon fibers effectively restrained crack propagation by providing bonding and mechanical interlocking across crack surfaces. Similarly, Maia et al. [21] showed that the incorporation of coir fibers significantly improved the flexural strength and fracture toughness of cement mortar, although a slight reduction in elastic modulus and compressive strength was observed. In addition to improving mechanical performance, fibers have also been shown to enhance the durability and deformation resistance of cementitious materials. Xue et al. [22] reported that the combined use of graphite tailings and basalt fibers effectively improved both the mechanical properties and drying shrinkage behavior of cement mortar. The optimum mixture, containing 20% graphite tailings and 0.1% basalt fibers, increased the compressive and flexural strengths by 13.89% and 14.08%, respectively. Furthermore, Lei et al. [23] employed DIC and acoustic emission techniques to investigate the crack propagation behavior of steel fiber-reinforced concrete. Their results revealed that steel fibers significantly enhanced fracture toughness, delayed crack propagation, and promoted a transition from brittle to ductile failure, with the crack-control effect becoming more pronounced as the fiber content increased. Furthermore, in addition to being directly used as structural materials, fiber-reinforced cement mortar also has potential as textile-reinforced mortar, which can be combined with fiber textiles for application in the strengthening and repair of masonry and concrete structures. Mazzuca et al. [24] investigated the tensile and bond properties of PBO-FRCM composites at elevated temperatures and found that high temperature significantly degrades the fiber-matrix interfacial bond performance, with the degree of degradation closely related to fiber type and temperature level. On this basis, Ombres et al. [25] further studied the residual bond behavior of FRCM-masonry interfaces after high-temperature exposure, revealing differentiated bond degradation laws for different fiber types of FRCM under high temperatures. The research indicates that the performance of the fiber-reinforced mortar matrix significantly affects the overall service behavior of textile-reinforced mortar strengthening systems. Therefore, conducting research on the mechanical properties and crack propagation of fiber-reinforced cement mortar has a clear engineering application background. Thus, incorporating an appropriate dosage of fibers into cement mortar helps to enhance its mechanical properties, retard crack propagation, improve structural stability and durability, and also endows it with potential as textile-reinforced mortar.

In recent years, first-principles calculations and other molecular-level simulation approaches have provided new avenues for understanding the mechanical behavior of phases in cement-based materials at the atomic scale. Mai et al. [26] systematically investigated the elastic and thermodynamic properties of the major clinker phases of Portland cement using density functional theory and revealed the intrinsic mechanical response mechanisms of each phase at the atomic scale. Hoang et al. [27] investigated the thermodynamic and mechanical properties of xonotlite and wollastonite in high-temperature geothermal well cement using first-principles calculations, and found that wollastonite exhibits a higher mechanical strength (97 GPa) and a lower coefficient of thermal expansion than xonotlite (66 GPa). The above studies indicate that molecular-level simulations can effectively supplement macroscopic experiments and provide theoretical support for elucidating the phase origins of the mechanical properties of cement-based materials. Currently, most research on fiber-reinforced cement materials focuses on the effects on macroscopic mechanical properties, conducting macroscopic mechanical property tests by incorporating carbon fibers, polypropylene fibers, basalt fibers, steel fibers, etc. [28,29,30,31]; however, certain research limitations remain. Firstly, most studies have focused on intact defect-free specimens, and the fiber reinforcement mechanism and crack propagation behavior of cement mortar containing prefabricated holes as initial defects have not been sufficiently investigated. Secondly, existing analyses mainly concentrate on the quantification of macroscopic mechanical indices, while the evolution characteristics of the mesoscopic strain field and the laws of crack initiation, propagation, and penetration remain inadequately addressed. Third, for the hybrid system of steel and carbon fibers, the combined strengthening effect at the micro- and macro-scales, as well as the optimal content matching under defective conditions, still lacks systematic experimental investigation. Based on the above research background, this study takes cement mortar with single-hole and double-hole prefabricated defects as the research object. Steel fibers alone, carbon fibers alone, and a hybrid of both fibers are incorporated, and uniaxial compression and splitting tensile tests are conducted in combination with DIC. From multiple perspectives, including macroscopic mechanical properties, mesoscopic strain evolution, and failure morphology, the reinforcement mechanisms and crack regulation effects of different fiber systems on defective cement mortar are revealed. The combined action mechanism and optimal content range of the hybrid fibers are clarified, providing experimental evidence for the performance optimization and engineering application of fiber-reinforced cement mortar under defective conditions.

## 2. Materials and Methods

### 2.1. Materials

**Figure 1:**
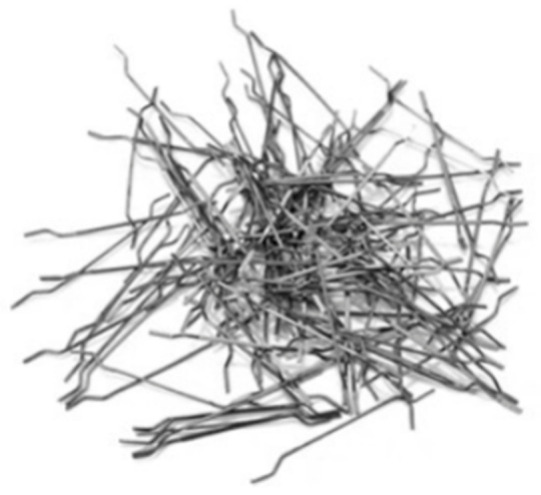
Hooked-end steel fiber.

The cement used in the test is P·O 42.5 ordinary Portland cement, and its basic composition is shown in Table 1. The sand is natural river sand, with a bulk density of 1460 kg/m^3^, a fineness modulus of 2.9, and is classified as medium sand. The mixing water was ordinary tap water from the laboratory. The reinforcing materials are steel fibers and carbon fibers, with their performance parameters shown in Table 2. The appearance of the steel fibers is shown in Figure 1. Hydroxypropyl methyl cellulose manufactured in China was used as the dispersant.

### 2.2. Specimen Design

**Figure 2:**
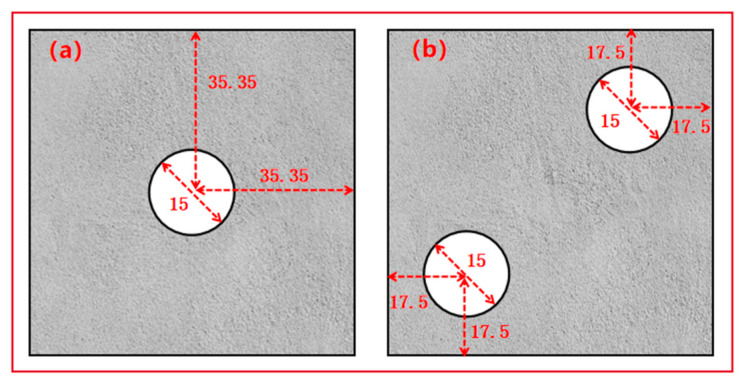
Prefabricated hole position diagram (mm): (**a**) single hole and (**b**) double hole.

To establish a model of material defects and damage, during the preparation of specimens, polyvinyl chloride (PVC) tubes of specific dimensions were embedded to create through-holes. The PVC tubes were withdrawn before the initial setting of the mortar, resulting in well-defined artificial holes in the specimens, thereby simulating the defect state of the material during actual service. As shown in Figure 2, the hole configuration scheme is divided into two types: single-hole specimens (with a through-hole of 15 mm in diameter set at the center of the specimen) and double-hole specimens (with two through-holes of 15 mm in diameter set at symmetrical positions in the specimen). The test mix proportions are shown in Table 3. The fiber content is expressed as a volume percentage of the specimen. The methyl cellulose content is a mass percentage of the carbon fibers. All specimens were prepared using a uniform procedure. The dispersant and water were first added to the mortar mixer and mixed uniformly. After the dispersant had dissolved, the carbon fibers were added and rapidly mixed to form a uniform fiber suspension. The weighed cement and river sand were then placed into the mixer and rapidly mixed for 3 min. For groups containing steel fibers, the steel fibers were uniformly sprinkled into the mixture during this stage, followed by an additional 3 min of mixing. Subsequently, the entire mixing water was uniformly added, and after 3 min of mixing, the fresh mortar was discharged and cast into the molds. For the compressive strength and splitting tensile strength tests, three cubic parallel specimens of 70.7 × 70.7 × 70.7 mm were prepared for each group. After being cured under standard conditions (temperature 20 ± 2 °C, relative humidity > 95%) for 28 days, the specimens were taken out for mechanical property tests.

### 2.3. Test Methods

**Figure 3:**
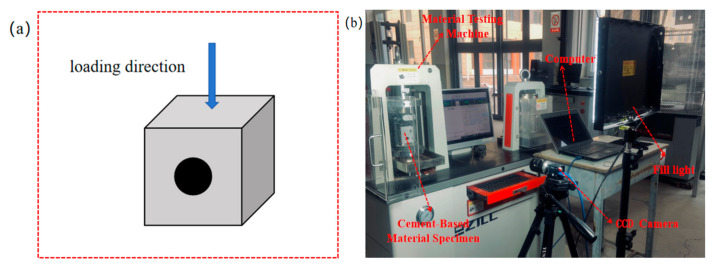
(**a**) Loading direction and (**b**) testing device.

Compressive and tensile tests were carried out on cement mortar specimens at loading rates of 0.5 kN/s and 0.2 kN/s. As shown in Figure 3a, the loading direction is perpendicular to the hole axis. During the tests, DIC was used to monitor the surface displacement field and strain field of the specimens. The test setup is shown in Figure 3b, where a 9 million pixel CCD camera manufactured by Daheng Image, Beijing, China is used for image acquisition, with the frame rate set to 12 frames/s and a pixel size of 2.5 μm. The DIC analysis region covered the entire surface of the specimen containing the holes, with a subset size of 29 × 29 pixels, a calculation step of 5 pixels, and a strain calculation window of 7 × 7 pixels. The zero-mean normalized cross-correlation function, which offers high reliability and noise immunity, was adopted for subset matching, and sub-pixel interpolation was employed to obtain a high-precision displacement field [32]. Before the test, the surface of the specimen to be tested was polished with sandpaper. After being smoothly polished, the specimen surface was sprayed with matte white paint. The paint was evenly sprayed from a distance of 30 cm from the specimen surface. After the white paint had air-dried, the same method was applied to spray matte black paint onto the surface to form a speckle pattern [33]. It is not advisable to spray the paint too thickly. Excessive thickness may affect crack initiation and propagation, while insufficient thickness may hinder observation by the DIC equipment [34].

## 3. Test Results and Discussions

### 3.1. Fiber-Reinforced Cement Mortar Mechanical Properties

#### 3.1.1. Compressive Strength

**Figure 4:**
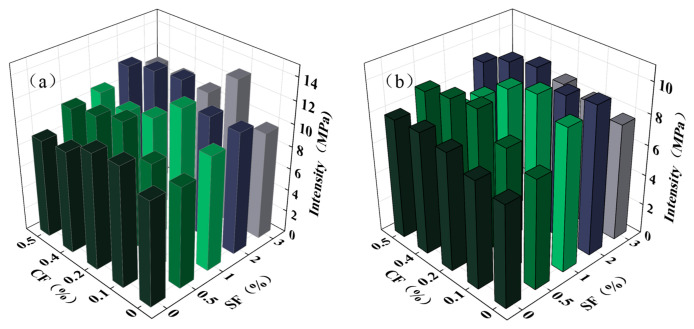
Compressive strength: (**a**) single hole and (**b**) double hole.

**Figure 5:**
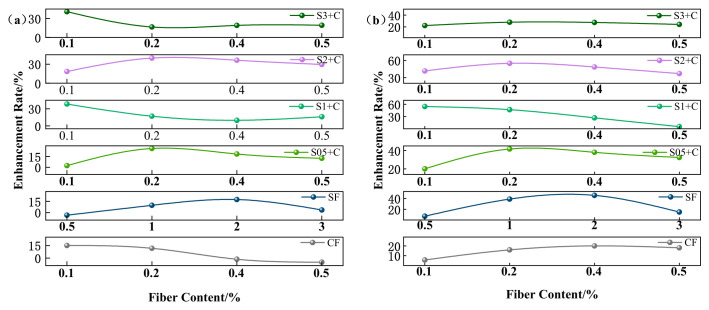
Compressive strength enhancement rate: (**a**) single hole and (**b**) double hole.

As shown in Figure 4 and Figure 5, the compressive strengths of the NM single-hole and double-hole specimens are 9.38 MPa and 6.70 MPa, respectively. The strength of the double-hole specimen is 28.6% lower than that of the single-hole specimen, indicating that a multi-defect structure significantly weakens the compressive performance of the material, with a more pronounced stress concentration effect under compression. Combined with the DIC strain contour map presented in Section 3.3.1, it can be observed that the edges of the holes are the primary strain concentration zones, where cracks preferentially initiate and propagate along oblique directions, exhibiting a typical brittle compression-shear failure. The compressive strength of specimens with steel fibers alone shows a trend of first increasing and then decreasing, reaching its peak at a fiber content of 2%, with enhancements of 17.38% and 45.82% for single-hole and double-hole specimens, respectively. DIC strain contour map analysis reveals that the incorporation of steel fibers significantly reduces the strain concentration zones, indicating that steel fibers can effectively retard crack propagation through the bridging effect and enhance structural integrity. However, when the fiber content is excessively high, non-uniform fiber distribution leads to local strain re-concentration, correspondingly resulting in a decline in strength, compromising the internal structural compactness of the mortar and weakening its compressive performance. The compressive strength improvement of specimens with carbon fibers alone is limited, with the enhancement mainly observed at low fiber content. At a fiber content of 0.1%, the enhancement is 15.03% for single-hole specimens and 15.97% for double-hole specimens. The carbon fibers can reduce the initial degree of strain concentration around the holes, resulting in a more uniform strain distribution and reducing the initiation of microcracks. However, rapid strain localization still occurs near the peak load, leading to the formation of through-going cracks, indicating a weak ability to control macroscopic crack propagation. The compressive performance of specimens hybridized with steel and carbon fibers is significantly improved, among which S2C02 exhibits the best performance, with compressive strengths of 13.10 MPa and 10.39 MPa for single-hole and double-hole specimens, respectively, corresponding to enhancement rates of 39.7% and 55.1%. The strain field distribution became more uniform after fiber incorporation. Stress concentration around the holes was significantly reduced. Cracks propagated in a more dispersed manner instead of forming concentrated through-cracks. From crack initiation to failure, crack propagation is greatly retarded. By bridging cracks around the holes and constraining matrix deformation, the hybrid fibers effectively alleviate the strength loss caused by multiple defects, demonstrating a favorable crack inhibition and structural reinforcement effect. Steel fibers suppress macroscopic crack propagation, while carbon fibers improve the microstructure. The combined effect of the two fibers can effectively ameliorate the stress distribution in the defective region, enhance the overall load-bearing capacity of the material, and increase its tolerance to defects.

Overall, the enhancement amplitude of the hybrid fiber system is more significant in double-hole specimens, which is consistent with the phenomenon of “weakening of the strain concentration band between the two holes” observed in the strain evolution and failure pattern, indicating that the hybrid fibers have a stronger ability to regulate complex stress fields. The improvement in compressive performance corresponds to the evolution of the strain field from a concentrated pattern to a diffused pattern and then to a uniform pattern. Furthermore, current analyses indicate that fibers improve compressive performance by regulating strain distribution, but their spatial distribution characteristics and coupling effect with the stress field remain insufficiently studied. In the future, further research can be conducted by combining 3D DIC and CT technology to establish a relationship model between the strain field and fiber distribution.

#### 3.1.2. Tensile Strength

**Figure 6:**
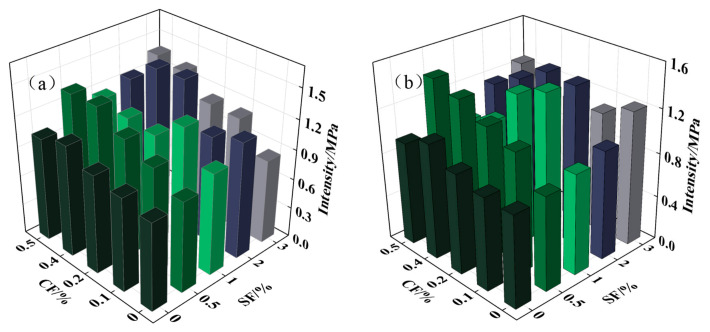
Tensile strength: (**a**) single hole and (**b**) double hole.

**Figure 7:**
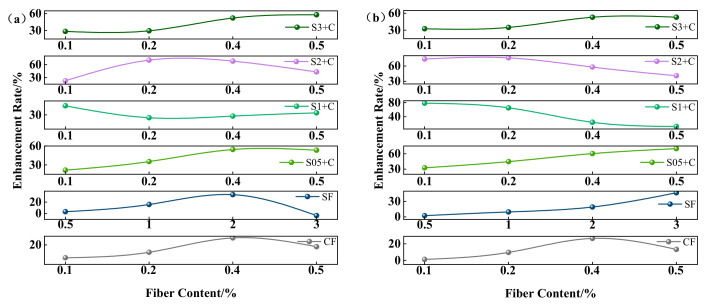
Enhancement rate of splitting tensile strength rate: (**a**) single hole and (**b**) double hole.

As shown in Figure 6 and Figure 7, the incorporation of fibers significantly enhances the tensile strength of cement mortar, but there are obvious differences in the reinforcement mechanisms among different fiber systems. Compared with the specimens without fibers, the splitting tensile strength of specimens with steel fibers alone shows a trend of “fluctuating increase”. At a fiber content of 2%, the strength of the single-hole specimen reaches 1.17 MPa, with an enhancement rate of 32.95%, representing the optimal value among the steel fiber-only group. At a fiber content of 3%, the strength of the double-hole specimen is 1.22 MPa, with an enhancement rate of 46.99%. Combined with the DIC strain contour map presented in Section 3.3.2, the steel fibers create a wide high-strain zone on both sides of the cracks, significantly reducing the crack propagation rate and suppressing crack opening, indicating that the steel fibers bear the tensile stress across the cracks through the bridging effect, thereby delaying crack penetration. The tensile strength of specimens with carbon fibers alone shows a relatively stable increase with the increase in fiber content. At a fiber content of 0.4%, the splitting tensile strength is enhanced by 27.27% and 26.51% for single-hole and double-hole specimens, respectively. The strengthening advantage of carbon fibers lies in their ultra-fine size, which allows them to be dispersedly distributed in the mortar matrix, hindering the initiation and early propagation of microcracks. Therefore, the strength improvement is more stable, but the overall enhancement magnitude is lower than the optimal level achieved with steel fibers alone. Combined with the DIC strain contour map analysis in Section 3.3.2, its main role is to reduce the degree of strain concentration around the holes and delay crack initiation, resulting in a more uniform strain distribution. However, after the formation of major cracks, they still penetrate relatively quickly, indicating that carbon fibers have a limited ability to constrain the crack propagation stage. The steel–carbon fiber hybrid specimens exhibit the best tensile performance. At a hybrid ratio of S2C02, the tensile strength reaches its peak value, with increases of 70.45% and 75.9%, respectively. Moreover, S05C02 also exhibits excellent tensile performance, with tensile strength increases of 63.64% and 61.45% for single-hole and double-hole specimens, respectively. When the steel fiber content is fixed at 2%, the enhancement rates at carbon fiber contents of 0.2%, 0.4%, and 0.5% all exceed 40%. Furthermore, this combination achieves a good balance between compressive and tensile performance, not only providing significant reinforcement but also using a relatively low carbon fiber content, which avoids the dispersion difficulties and increased cost associated with high fiber content. Combined with the DIC strain contour map analysis in Section 3.3.2, the strain field distribution of this type of specimen is continuous and uniform. The high-strain zone expands in range, but its concentration degree decreases, and the strain concentration zone is significantly reduced. This indicated that carbon fibers inhibit the initiation of microcracks, while steel fibers control the propagation of macroscopic cracks, and their combined effect achieves full-process regulation of cracking.

In general, under both compressive and tensile loading, the enhancement effect of the hybrid fiber system is more prominent in double-hole specimens, and the strain concentration zone is significantly weakened, indicating that it has higher crack stability under complex defect conditions. Furthermore, the existence of an optimal fiber content for each group indicates that the fiber reinforcement effect exhibits a clear matching relationship. This study reveals the regulatory effect of fibers on crack evolution from the perspective of the strain field, but a quantitative model for the synergistic mechanism of fibers at different scales is still lacking. In the future, a combination of multiscale simulation and experiments can be used to further investigate the influence of fiber dispersion and long-term service conditions on tensile performance.

### 3.2. Tensile-Compressive Strength Ratio

In current engineering practice, the ratio of tensile strength to compressive strength (tensile-to-compressive strength ratio) is widely used as an indirect evaluation indicator of the brittleness of cement-based materials, and its core significance lies in the fact that the ratio can, to a certain extent, reflect its crack resistance and load-bearing capacity. The calculation formula is shown in Equation (1). Generally, the lower the tensile-to-compressive strength ratio, the more intuitively it reflects the brittle characteristics of the material; such materials are prone to rapid crack propagation and sudden failure when under loading. In contrast, a higher ratio indicates superior crack resistance of the cement mortar and shows a trend of improved toughness [35,36].

(1) $$m=f_{ts}/f_{cc}$$

where *m* is the tensile-to-compressive strength ratio; $f_{ts}$ is the tensile strength of the specimen; $f_{cc}$ is the compressive strength of the specimen.

**Figure 8:**
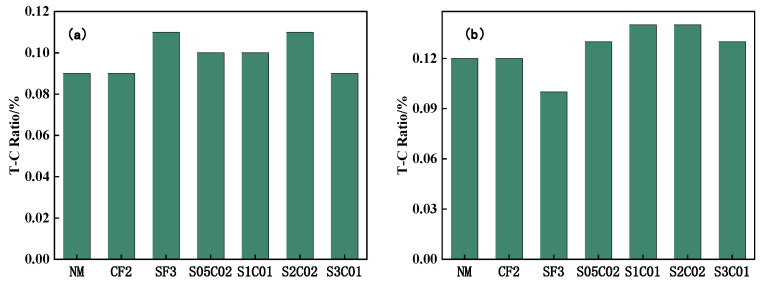
Tension–compression ratio: (**a**) single hole and (**b**) double hole.

As shown in Figure 8, the tensile-to-compressive strength ratio of the fiber-reinforced specimens is significantly better than that of the NM specimens. For single-hole specimens, S2C02 (0.115) is 22.34% higher than NM (0.094). For double-hole specimens, S2C02 (0.141) is 13.71% higher than NM (0.124). This result is fully consistent with the strain evolution and failure pattern of the cement mortar. After the incorporation of fibers, carbon fibers inhibit the initiation of microcracks in the specimens, while steel fibers suppress the propagation of macroscopic cracks, thereby prolonging the time from crack initiation to the stage of penetrating failure. Collectively, the incorporation of carbon fibers and steel fibers effectively improves the tensile-to-compressive strength ratio of cement mortar, enhances its crack resistance, and improves the failure mode of the material.

### 3.3. Failure Pattern

#### 3.3.1. Compression Condition

**Figure 9:**
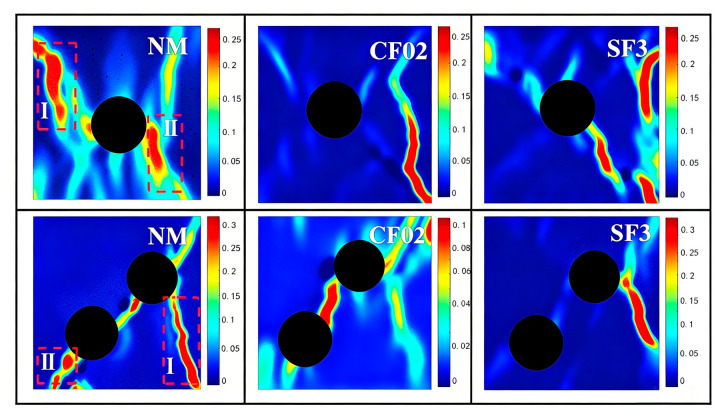
Compressive test strain contour map.

**Figure 10:**
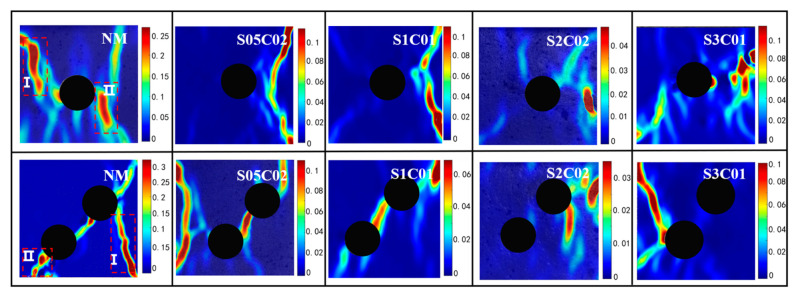
Compressive test strain contour map.

As shown in Figure 9 and Figure 10, under uniaxial compression, a significant stress concentration phenomenon occurs around the prefabricated holes, and there are obvious differences in the strain evolution and failure pattern of the specimens among different groups. For the NM specimens, strain is highly concentrated at the edges of the holes. In single-hole specimens, a through-going main crack forms along the oblique direction. In double-hole specimens, a strain concentration band develops between the two holes, evolving into a “y”-shaped failure. The overall behavior is characterized by sudden brittle compression-shear instability. In the figure, zones “I” and “II” indicate the locations of main crack propagation in the reference group. After the incorporation of steel fibers, the degree of strain concentration is significantly reduced, and the high-strain zone transitions from localized concentration to regional diffusion. The number of main cracks is markedly decreased, and the overall crack penetration process is delayed. A certain degree of strain accumulation occurs before specimen failure, indicating an improvement in ductility. However, when the fiber content is excessively high, strain localization still exists, which is consistent with the trend of compressive strength “first increasing and then decreasing”. In the early stage of loading, the strain distribution of specimens with carbon fibers alone is more uniform, and the stress concentration around the holes is partially relieved, delaying the initiation of microcracks. However, when approaching the peak load, the strain still rapidly localizes and develops into through-going cracks, and the macroscopic failure remains predominantly brittle.

In contrast, the strain field of the steel–carbon hybrid specimens is the most uniform overall, achieving a combined complementarity between microcrack suppression and macroscopic crack bridging. The high-strain zones exhibit a diffuse distribution, the stress concentration induced by the holes is significantly weakened, and the cracking pattern shifts from concentrated through-going failure to the development of multiple dispersed cracks, with the difficulty of crack penetration being significantly increased. Represented by S2C02, the strain development process is gradual, with clear deformation precursors before failure, exhibiting a transition from brittle to quasi-ductile behavior. As shown in zones I and II, after the incorporation of fibers, the number of main cracks is significantly reduced, and the strain distribution gradually evolves from a “concentrated” pattern to a “diffused” pattern and then to a “uniform” pattern, with hybrid fibers exhibiting the most significant effect in weakening the hole effect and improving structural stability.

#### 3.3.2. Tension Condition

**Figure 11:**
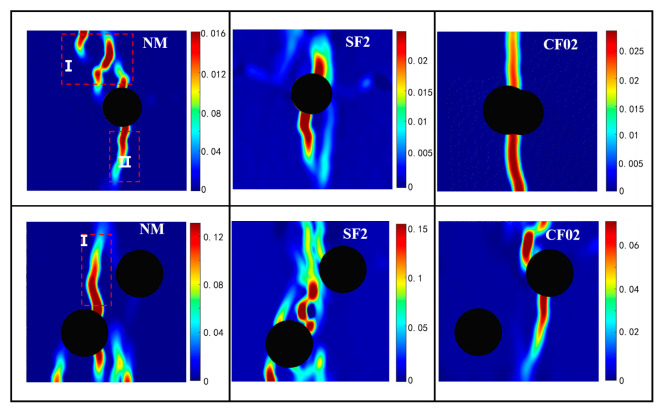
Tensile test strain contour map.

**Figure 12:**
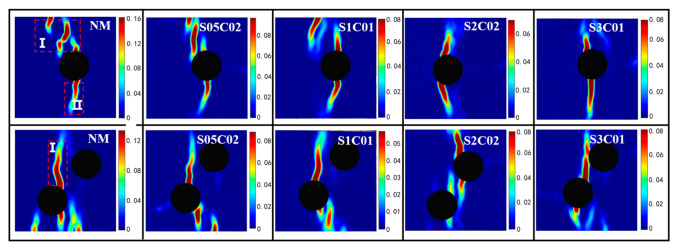
Tensile test strain contour map.

As shown in Figure 11, in the splitting tensile test, the strain evolution and failure pattern of the specimen surface obtained by DIC monitoring exhibit stress concentration characteristics similar to those observed in the compressive test. However, due to the different loading modes, there are significant differences in strain distribution, crack propagation path, and failure mode. The strain distribution and crack propagation path are mainly governed by tensile stress, with cracks developing perpendicular to the loading direction and stress concentration occurring at the holes. In the figure, zones “I” and “II” indicate the locations of main crack propagation in the reference group. In the NM group, strain is highly concentrated at the edges of the holes and along the central axis of the specimen. Once cracks initiate, they propagate rapidly through the specimen, exhibiting typical splitting brittle failure without an obvious strain propagation stage. In the SF group, steel fibers retard crack propagation through the bridging effect across cracks and form a larger strain distribution zone on both sides of the cracks, indicating that the bridging effect of steel fibers is significant under tensile conditions. In the CF group, specimens with carbon fibers alone can effectively inhibit the initiation of microcracks. From the strain contour map, it is clearly observed that the final failure pattern of the specimen exhibits only one main crack, which effectively hinders the initiation and propagation of microcracks. However, after the main crack forms, it still penetrates relatively quickly, indicating a limited ability to control macroscopic crack propagation. As shown in Figure 12, the steel–carbon hybrid specimens exhibit the best strain regulation effect: the strain field distribution is uniform and continuous, the high-strain zone expands in range, but its peak value decreases, the crack propagation path becomes more dispersed, the width of the main crack is significantly reduced, and some specimens show a feature of cooperative development of multiple cracks. Taking S2C02 as a representative, crack penetration is significantly delayed, the failure process is gradual, and the crack resistance is greatly improved.

### 3.4. Analysis of Fiber Inhibition Effect Based on Crack Propagation Time

**Figure 13:**
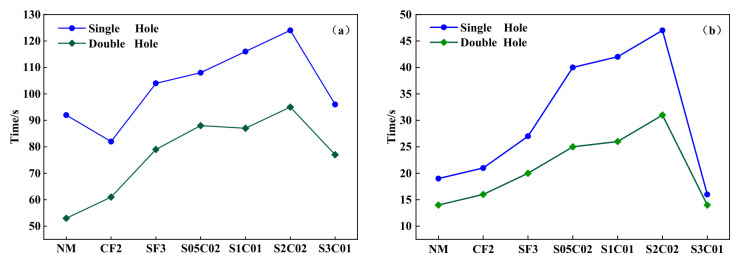
Time from crack initiation to through-and-through failure: (**a**) compression test and (**b**) tensile test.

Using the time from crack initiation to penetrating failure as a quantitative indicator, combined with DIC strain field analysis of the influence of fibers on crack propagation in cement mortar, the results showed that the crack propagation behavior is highly correlated with the mechanical properties and failure pattern of the specimens. Under the influence of stress concentration at the prefabricated holes, the NM group has no crack-restraining capability, and cracks initiate and propagate rapidly. For the compressive condition, the crack propagation times for single-hole and double-hole specimens are 92 s and 53 s, respectively; for the tensile condition, they are 19 s and 12 s, respectively. The stress concentration is more pronounced in the double-hole specimens, leading to faster crack propagation, which is the fundamental reason for their poor mechanical properties and brittle failure. Single fiber incorporation has stage-specific limitations in crack regulation. The addition of carbon fibers alone (CF02) can only inhibit microcrack initiation, but has a weak restraining effect on macroscopic cracks. Under tensile conditions, the crack propagation times for single-hole and double-hole specimens increase to 21 s and 24 s, respectively; under compressive conditions, the time for the single-hole specimen instead decreases to 82 s. This is consistent with the characteristics of a slight increase in tensile strength and limited improvement in compressive strength. The addition of steel fibers alone (SF2) delays macroscopic crack propagation through the bridging effect. Under compressive conditions, the crack propagation times for single-hole and double-hole specimens are 104 s and 79 s, respectively; under tensile conditions, they are 25 s and 33 s, respectively. The increase is more significant for double-hole specimens, corresponding to a higher strength improvement of the double-hole specimens. However, the regulation of microcrack initiation is insufficient, and there is an upper limit to the improvement in ductility. As shown in Figure 13, the steel–carbon hybrid fibers achieve full-process combined regulation of microcrack initiation inhibition and macroscopic crack bridging, greatly increasing the crack propagation time, and the regulation effect is more prominent for the complex defects of double-hole specimens. For S2C02, under compressive conditions, the crack propagation times for single-hole and double-hole specimens reach 124 s and 95 s, respectively; under tensile conditions, they reach 47 s and 45 s, respectively. Carbon fibers reduce microcrack initiation at the source, while steel fibers constrain further propagation of macroscopic cracks. Their synergy makes the strain field distribution of the specimens more uniform, and cracks propagate in a more dispersed manner, corresponding to the optimal mechanical properties and the transition of failure mode from brittle to quasi-ductile. However, for S3C01, the excessively high fiber content leads to non-uniform distribution and compromised matrix compactness, causing the crack regulation effect to decline, which is consistent with the trend of a significant decline in the mechanical properties of this group. Overall, the crack propagation time of the specimens is highly positively correlated with the enhancement rate of mechanical properties and the ductility of the failure mode. The stronger the full-process regulation capability of fibers on cracking, the longer the time from crack initiation to penetration, and the more significant the improvement in material performance. This also confirms that the fiber reinforcement effect exhibits an optimal content matching relationship rather than a simple superposition of dosages, and that the combined effect of the hybrid system is the core factor in enhancing the crack resistance of defective cement mortar.

## 4. Conclusions

(1) Both steel fibers and carbon fibers improved the mechanical performance and crack resistance of cement mortar containing prefabricated defects. Steel fibers mainly enhanced strength and restrained macrocrack propagation through a bridging effect, whereas carbon fibers primarily inhibited microcrack initiation and improved strain distribution. The hybrid steel–carbon fiber system exhibited a pronounced combined effect, with the S2C02 mixture providing the best overall performance in terms of strength, toughness, and crack resistance.

(2) DIC analysis revealed that fiber incorporation significantly altered the strain evolution and failure behavior of defective cement mortar. Hybrid fibers reduced strain concentration around the defects, promoted a more uniform strain distribution, delayed crack localization, and transformed the failure mode from brittle to quasi-ductile.

(3) The crack propagation time increased significantly after fiber incorporation and was positively correlated with the improvement in mechanical properties and toughness. The hybrid steel–carbon fiber system showed the most effective crack-control capability, demonstrating that the combined interaction between microcrack suppression and macrocrack bridging is the key mechanism for enhancing the defect tolerance of cement mortar.

(4) It is clarified that the hybrid fibers exhibit more significant reinforcement and crack regulation effects under complex defect conditions with double holes, with a more pronounced mitigation of stress concentration, thereby enriching the conclusions regarding the applicable scenarios of fiber reinforcement for defective cement-based materials.

## Figures and Tables

**Table 1:** Main chemical components of cement.

$SiO_{2}$	${Al}_{2}O_{3}$	$Fe_{2}O_{3}$	CaO	MgO	${SO}_{3}$	$K_{2}O$	$Na_{2}O$
21.91	5.36	3.8	64.3	1.6	2.07	1.39	0.26

**Table 2:** Fiber performance parameters.

Fiber Types	Density/ g∙cm^−3^	Diameter/ mm	Length/ mm	Tensile Strength/ MPa	Elastic Modulus/ GPa	Aspect Ratio	Manufacturer
Hooked-End Steel Fibers	7.85	0.75	30	1100	200	40	Harex Shanghai, China
Carbon Fibers	1.8	0.007	15	4900	230	2140	Torayca Tokyo, Japan

**Table 3:** Trial mix proportion.

Number	CF/%	SF/%	Cement/ kg·m^−3^	Sand/ kg·m^−3^	Water/ kg·m^−3^	Dispersant/ %
NM	0	0	300	1460	270	0
SF05	0	0.5	300	1460	270	0
SF1	0	1	300	1460	270	0
SF2	0	2	300	1460	270	0
SF3	0	3	300	1460	270	0
CF01	0.1	0	300	1460	270	0.1
CF02	0.2	0	300	1460	270	0.1
CF04	0.4	0	300	1460	270	0.1
CF05	0.5	0	300	1460	270	0.1
S05C01	0.1	0.5	300	1460	270	0.1
S05C02	0.2	0.5	300	1460	270	0.1
S05C04	0.4	0.5	300	1460	270	0.1
S05C05	0.5	0.5	300	1460	270	0.1
S1C01	0.1	1	300	1460	270	0.1
S1C02	0.2	1	300	1460	270	0.1
S1C04	0.4	1	300	1460	270	0.1
S1C05	0.5	1	300	1460	270	0.1
S2C01	0.1	2	300	1460	270	0.1
S2C02	0.2	2	300	1460	270	0.1
S2C04	0.4	2	300	1460	270	0.1
S2C05	0.5	2	300	1460	270	0.1
S3C01	0.1	3	300	1460	270	0.1
S3C02	0.2	3	300	1460	270	0.1
S3C04	0.4	3	300	1460	270	0.1
S3C05	0.5	3	300	1460	270	0.1

Note: NM denotes plain cement mortar; SF and CF denote the addition of steel fibers alone and carbon fibers alone, respectively; S and C represent steel fibers and carbon fibers in the hybrid system; and 01, 02, 04, 05, 1, 2, and 3 represent fiber contents of 0.1%, 0.2%, 0.4%, 0.5%, 1%, 2%, and 3%, respectively.

## Data Availability

The original contributions presented in this study are included in the article. Further inquiries can be directed to the corresponding author.

## Abbreviations

The following abbreviations are used in this manuscript:

CF	Carbon Fiber
NM	Normal Mortar
SF	Steel Fiber
DIC	Digital Image Correlation Technology
